# Molecular Characterization of the Chicken Parvovirus Based on VP1 Gene Circulating in Brazilian Chicken Flocks

**DOI:** 10.3390/microorganisms12061065

**Published:** 2024-05-24

**Authors:** Luis F. N. Nuñez, Silvana H. Santander-Parra, Claudete S. Astolfi-Ferreira, Anthony Loor-Giler, Antonio J. P. Ferreira

**Affiliations:** 1 Facultad de Ciencias de la Salud, Carrera de Medicina Veterinaria, Universidad de Las Américas, Antigua Vía a Nayon S/N, Quito 170124, Ecuador; silvanahsp@yahoo.com; 2 One Health Research Group, Universidad de Las Americas, Quito 170124, Ecuador; 3 Avian Pathology Laboratory, Department of Pathology, College of Veterinary Medicine, University of São Paulo (USP), Av. Prof. Dr. Orlando M. Paiva, 87, São Paulo 05508-270, Brazil; csastolfi@gmail.com (C.S.A.-F.); ajpferr@usp.br (A.J.P.F.); 4 Laboratorios de Investigación, Dirección General de Investigación, Universidad de las Américas (UDLA), Antigua Vía a Nayón S/N, Quito 170124, Ecuador; a.abel.loor.giler@gmail.com; 5 Facultad de Ingeniería y Ciencias Aplicadas, Carrera de Ingeniería en Biotecnología, Universidad de Las Américas (UDLA), Antigua Vía a Nayón S/N, Quito 170124, Ecuador

**Keywords:** enteric diseases, chicken parvovirus, ABU-P1, genotyping

## Abstract

Parvovirus infection affects several animal species, especially young animals. In birds, parvovirus infection has been described in Muscovy ducks, turkeys, and chickens, all of which had enteric diseases characterized by diarrhea. Chicken parvovirus (ChPV) has been detected in poultry around the world in animals affected by enteric problems, showing dwarfism, cloacal pasting, and diarrhea. In Brazil, ChPV was detected in chickens affected by diarrhea fifteen years ago. However, the genetic characteristics of ChPV circulating in chicken flocks were not determined. Therefore, the aim of the present investigation was to determine the genetic characteristics of the VP1 gene from ChPV detected in chickens affected by enteric diseases in Brazil. For this purpose, a molecular approach was used. Specific primers were designed to flank the complete VP1 gene of ChPV and amplify it using PCR. The amplified products from samples of chickens with enteric diseases were sequenced, and 22 complete CDs of the VP1 gene were obtained. These samples, compared to the ABU-P1 sequence, showed 17 sequences with high nucleotide (NT) similarity of 92.7–97.4% and amino acid (AA) similarity of 94.8–99.5% associated with Runting and Stunting syndrome (RSS); there were also five samples associated with hens with diarrhea with unusual jejunal dilatation (JD) that had less similarity than the RSS sequences (NT of 86.5% and AA of 93–93.1%). The phylogenetic analysis determined four groups. Group I had sequences from Korea. The second group included sequences from Korea, China, and Brazil (not included in this work). The third group had studied RSS sequences grouped with the ABU-P1 strain and sequences from China and the United States. Finally, the sequences from JD were clustered in a separate group with a bootstrap of 100%, a group that was denoted as group IV, and included sequences from China. RDP4 and SimPlot analysis showed one point of recombination with the sequences of group III ChPV in the JD sequences. Herein, we show that circulating strains of ChPV exhibit genetic differences in the VP1 gene in Brazilian chicken flocks. Nevertheless, more studies are needed to determine the probability of a new genetic group of ChPV based on the analysis of the complete genome.

## 1. Introduction

Enteric diseases cause important problems in avian health. Three syndromes have been described in poultry: runting and stunting syndrome (RSS) in chickens and poult enteritis complex (PEC) and poult enteritis mortality syndrome (PEMS) in turkeys [1]. Several viruses have been identified as being associated with these syndromes, including rotaviruses, astroviruses, reoviruses, and parvoviruses [2,3,4,5]. The latter virus was diagnosed in chickens affected with RSS, principally in young animals. Chicken parvovirus (ChPV) is a DNA virus approximately 5.1 kB in length [6,7,8]. The genomic organization of ChPV includes two open reading frames (ORFs) that encode nonstructural (NS) and structural proteins (VP1 and VP2); ORF1 includes the NS protein, and ORF2 includes structural proteins [1,6].

ChPV was detected for the first time by Kisary in 1984, who described the presence of the virus in chicks affected with diarrhea. This virus produces ruffled feathers, cloacal filling, apathy, depression, dwarfism, and mortality [9,10]. Experimental studies with ChPV reproduced the disease, showing clinical signs of RSS, and the pathological findings observed in naturally infected animals were characterized by intestines filled with liquid, gas bubbles, and nondigested food [11,12,13]. The microscopic lesions vary between the presence of cystic enteritis or the absence of any cellular lesion [12,13]. ChPV was first detected using electronic microscopy (EM), showing few viral particles in the enteric content of affected chicks [10,11]. In the following years, other diagnostic methods were used for ChPV detection, such as immunofluorescence [11], ELISA [14], immunohistochemistry [13], and molecular techniques currently used for viral DNA detection [15,16,17,18]. Most molecular tests for ChPV detection amplify a part of the NS gene [16,18,19]. Using these molecular assays, ChPV was detected in Europe [17,20], Asia [21,22,23], and America [2,7,16,17,20,21,24], determining that the virus has a global distribution. However, knowledge about the genetic characteristics of ChPV is limited. The first information on the ChPV genome was the characterization of the NS gene, which showed that ChPV and TuPV were closely related to each other and were representative of a new member of the parvovirus family [25]; this allowed an endpoint PCR assay to be developed that amplifies 561 bp of the gene [18]. Many studies were carried out using this part of the genome, with high similarity in the percentage of amino acids and nucleotides between the analyzed sequences [2,16,17,18,20,25,26,27]. In the last ten years, many complete genomes of ChPV have been published. In Korea, three novel complete genomes of ChPV associated with RSS had differences compared to the reference strain ABU-P1 [8]. In the United States, other complete genome sequences of ChPV from chickens with RSS were related to ABU-P1 and Korean strains. Additionally, the VP1 gene was described as a specific host determinator between chickens and turkeys [24]. Both studies used SANGER technology. Next-generation sequencing (NGS) has also been used and has generated some complete genome sequences of ChPV, principally from Asia [7], but novel genotypes of viruses have not been reported.

In some countries, as well as in Brazil, there is widespread information about the association of ChPV with enteric diseases. However, the virus is related to other pathologies in addition to enteric disease, such as cerebellar hypoplasia and hydrocephalus reported in young chickens showing neurological signs, impaired mobility, and diarrhea [28]. Similarly, in Brazil, ChPV was detected in chickens affected with RSS and in white laying hens with enteric disease with unusual jejunal dilatation and high mortality, extreme weight loss, apathy, and diarrhea; despite this, there is a lack of knowledge as to what genotype of ChPV is circulating in Brazilian chicken flocks. For this reason, the aim of the present work was to determine the genetic features of the VP1 gene of ChPV circulating among commercial chicken flocks in Brazil.

## 2. Material and Methods

### 2.1. Samples

In the present work, 22 samples from the enteric tract of chickens suffering digestive problems were used. From these samples, seventeen came from chickens affected with RSS, and the remaining five came from hens affected with enteric diseases and showing unusual intestinal dilatation. All samples were used in accordance with the guidelines and the approval of the Committee on the Care and Use of Laboratory Animal Resources of the School of Veterinary Medicine, University of Sao Paulo, Brazil, under protocol number #2569/2012. The samples were previously analyzed by molecular assays for enteric viruses and were PCR positive for ChPV (Table 1). These samples were subjected to a molecular approach to determine the genetic features of the complete VP1 gene and to characterize the studied virus. All the analyses were performed using molecular and bioinformatics tools as described below.

### 2.2. Nucleic Acid Extraction

Nucleic acid (DNA) was extracted from 100 mG of intestine using TRIzol (Invitrogen by Life Technologies, Carlsbad, CA, USA) reagent according to the manufacturer’s instructions.

### 2.3. PCR for VP1 Gene Amplification

Amplification of the VP1 gene was performed using conventional PCR. For this purpose, a pair of primers was designed using Geneious version 11.1.4 based on the reference sequence ABU-P1 (NC_024452.1) and other sequences from complete genomes of ChPV available in GenBank (KJ486489.1; KM254173.1; KM598414.1; KM598416.1; KU523900.1). The PCR used 23 µL of reaction mixture with 0.5 µM of the designed forward and reverse primers (Table 2), 2X Buffer, 5 mM of each dNTP, 37.5 mM of Mg, 1 U of Platinum Taq DNA polymerase (Thermo Fisher Scientific, Carlsbad, CA, USA), and 2 µL of DNA. The samples were subjected to PCR amplification under the following conditions: one cycle at 95 °C for 5 min to completely denature the DNA; 35 cycles at 95 °C for 30 s for template denaturation, 60 °C for 30 s for primer annealing, and 72 °C for 45 s for extension; and a final extension step at 72 °C for 10 min. The reaction was maintained at 4 °C until stored at −20 °C. The PCR products were subjected to electrophoresis on a 1% agarose gel.

### 2.4. Cloning and Sequencing of the Complete VP1 Gene

The amplified product with the designed primers was purified using CleanSweep PCR Purification (Thermo Fisher Scientific, Carlsbad, CA, USA) as described by the manufacturer. To sequence the purified product, the product was ligated into the pCR 2.1-TOPO^®^TA Cloning Kit (Thermo Fisher Scientific, Carlsbad, CA, USA) according to the manufacturer’s instructions. The plasmid was transformed into E. coli chem competent cells according to the manufacturer’s instructions. Three colonies were cultured in 3 mL of Luria Bertani (LB) broth and shaken at 230 rpm for 20 h. Plasmid DNA was extracted from the LB broth bacterial suspension using a QIAprep Spin Miniprep Kit (Qiagen, CA, USA). The plasmid DNA was sequenced in the forward and reverse directions using a BigDye^®^ Terminator v3.1 Cycle Sequencing kit (Applied Biosystems by Thermo Fisher Scientific, Carlsbad, CA, USA) using M13 forward and reverse primers. To target all length sequences of the complete VP1 gene, a primer walking strategy was applied, sequencing approximately 2.3 Kb. The obtained electropherograms were analyzed using the Geneious 11.1.4 package software using the novo assembly, and the complete VP1 gene was also predicted in the software. The consensus-obtained sequences of the complete VP1 gene were aligned and compared with other sequences of ChPV available in GenBank using the CLUSTAL W method in ClustalX version 2.0.11 Package software (European Bioinformatics Institute, Saffron Walden, UK). The similarity of nucleotides (NT) and amino acids (AA) was determined using Bioedit version 7.1.3, and the analysis of the complete gene coding NT sequences versus the VP1 gene of ABU-P1 was performed using the Kimura two-parameter model in the SimPlot version 3.5.1 software package. The phylogenetic tree was built using the neighbor-joining statistical method and Tamura-Nei substitution model with 1000 bootstrap replicates in the MEGA 7 software package [29].

### 2.5. Recombination Analysis

The sequences obtained herein were tested for the presence of recombination events using the recombination detection program (RDP4) version v.4.43 and the SimPlot version 3.5.1 software package. The complete sequences of the VP1 gene from ChPV available in GenBank were included in the RDP4 analyses, incorporating the reference sequences of ChPV (NC_024452.1) and TuPV (NC_024454.1). Different methods implemented in RDP4 version v.4.43 software were used to detect recombinant events under default settings for the different detection programs.

### 2.6. GeneBank Accession Numbers

The sequences of the complete VP1 gene of ChPV were submitted to GeneBank and were denoted by the accession numbers as follows: USP 93 (MW331564.1); USP 162 (MW331563.1); USP 238-1 (MW331565.1); USP 238-5 (MW331562.1); USP 259-10 (MW331561.1); USP 259-11 (MW331560.1); USP 336-7 (MW331559.1); USP 336-15 (MW331553.1); USP 336-16 (MW331551.1); USP 345-15 (MW331552.1); USP 358-7 (MW331550.1); USP 358-10 (MW331549.1); USP 362-7 (MW331548.1); USP 400-7 (MW331547.1); USP 401-3A (MW331546.1); USP 507-1 (MW331557.1); USP 507-3 (MW331556.1); USP 507-9D (MW331555.1); USP 507-20 (MW331558.1); USP 507-24 (MW331554.1); USP 710-1 (MW331545.1); USP 711-11 (MW331566.1).

## 3. Results

### 3.1. PCR for VP1 Gene Amplification and Sequencing

The designed primers amplified a product of 2248 bp. The product of each sample was cloned and transformed into E. coli chem competent cells. Three DNA plasmids from each sample were subjected to sequencing, and the obtained sequences were assembled with the de novo method. In the consensus sequence, an ORF was predicted, and a sequence with a length of 2028 bp that included a complete VP1 gene of ChPV was obtained. The sequences were analyzed with the BLAST tool, showing uniquely high similarity with sequences of ChPV and no similarity with sequences of any other organism. A total of twenty-two sequences of the complete VP1 gene were generated, five from cases of JD and seventeen from cases of RSS (Table 1).

The obtained sequences were aligned with other sequences available in GenBank, and the similarity of NT and AA was determined. The similarity matrix showed that the sequences studied fell into two groups: a group with sequences related to RRS and another group with sequences of ChPV related to JD.

The RSS sequences had a high similarity of NT (99.8–90.5%) and AA (100–93.4%) among them, and the JD sequences had a high similarity of NT (100–99.9%) and AA (100–99.8%). Comparing both groups to the reference sequence of ChPV ABU-P1 (NC_024452.1) showed that the RSS sequences had high similarity to NT (97.4–92.7%) and AA (99.5–94.8%) and the JD sequences had similarity to NT (86.5%) and AA (93.1–93%) (Table 3), similarity that was lower than the RSS sequences.

Therefore, when the sequences of both groups of ChPV obtained from cases of RSS and from cases of JD were compared, they showed a similarity of NT of 91.4–86% and of AA of 94.6–91.7%, exhibiting a difference in the similarity of NT and AA of the VP1 gene, with the difference being greater in the percentage of NT than in the percentage of AA.

### 3.2. Phylogenetic Analysis

The phylogenetic analysis showed that all sequences obtained herein were grouped as part of the ChPV group, and four well-defined groups were observed within this group.

**Figure 1:**
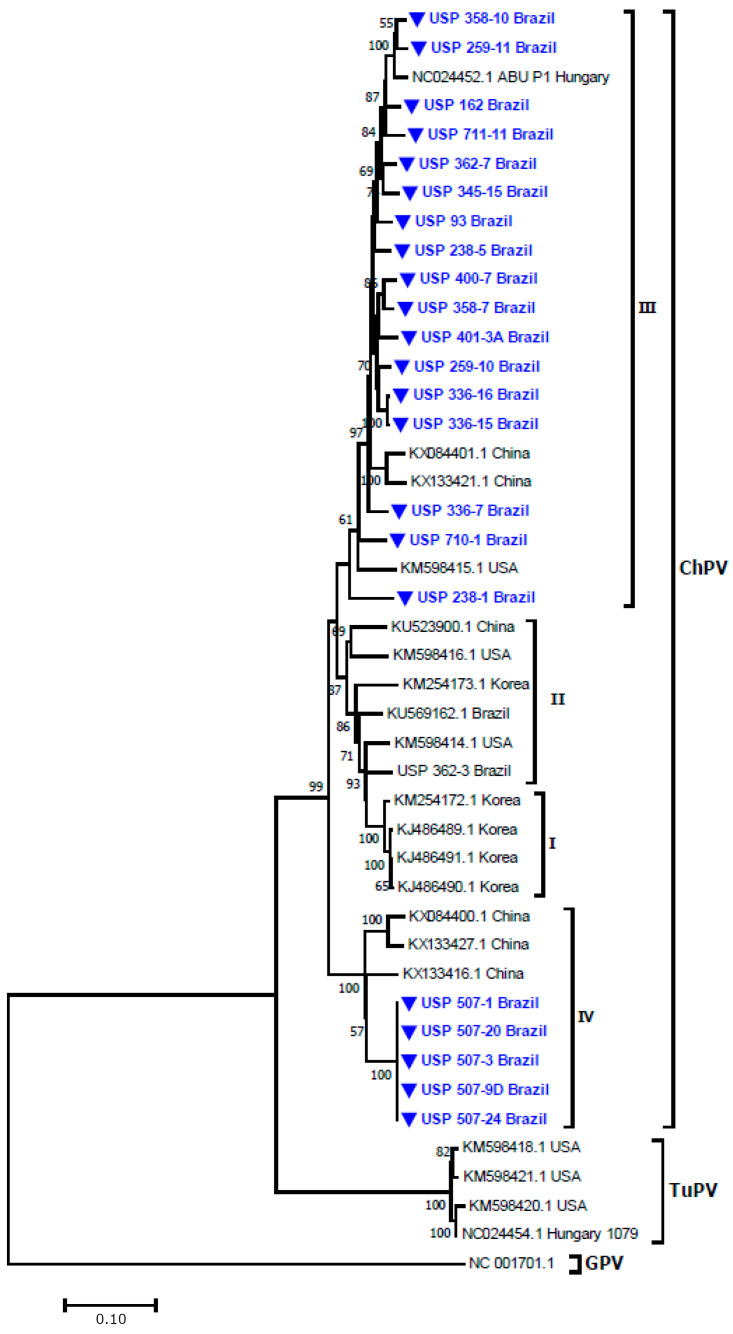
Phylogenetic analyses between sequences herein obtained of ChPV related to outbreaks of RSS and JD and other sequences of ChPV based on complete VP1 gene nucleotide sequences. Sequences were aligned using CLUSTAL W method in ClustlX 2 2.1. The phylogenetic tree was constructed using the MEGA 7 software package. The numbers along the branches refer to the bootstrap values of 1000 replicates. The scale bar represents the number of substitutions per site. Goose Parvovirus (GPV) was used as the out-group. The sequences obtained here are shown in blue. USP 362-3 Brazil (KM598415.1). TuPV = Turkey parvovirus. Korea = Republic of Korea.

The first group was formed by sequences from Korea associated with outbreaks of RSS with a bootstrap of 100%. The second group was formed with sequences from Korea, China, United States, and Brazil (KU569162.1 and KM598415.1) with a bootstrap of 87%. The third group included sequences herein studied from animals affected with RSS clustered with the reference sequence of ChPV ABU-P1 from Hungary and with sequences from the United States and China with a bootstrap of 61%. The fourth group included sequences from animals with enteric disease showing dilatation of the jejunum clustered in a separated group with a bootstrap of 100% (Figure 1). This group also included sequences from China. None of the sequences investigated here were clustered with the sequences of TuPV.

The results obtained here showed that Brazilian chicken flocks are circulating strains that are highly similar to European (reference strain of ChPV), American (United States), and Asian (Republic of Korea and China) sequences of ChPV, and we identified a genetic group of ChPV that was found in layer hens showing enteric disease characterized by dilatation of the jejunum.

### 3.3. Recombination Analysis

The recombination analyses of the sequences obtained here with RDP4 version v.4.43 and SimPlot version 3.5.1 software showed that the group of ChPV sequences from chickens with JD has one site of recombination, but for the group of sequences from cases of RSS, no site of recombination appears. The recombination events detected in RDP4 in the JD sequences were supported by several statistical measurement recombination assays, including RDP, GENECONV, BootScan, Maxchi, Chimera, SiScan, and 3Seq.

**Figure 2:**
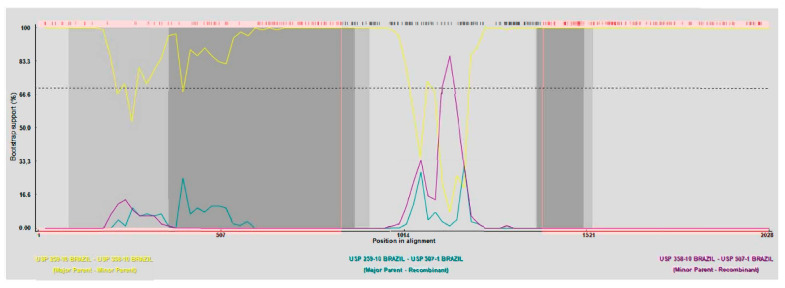
Graph of Bootscan analysis of sequences of ChPV here obtained compared with other sequences of ChPV in RDP4 version v.4.43. All sequences used for building a phylogenetic tree were used to determine points of recombination along with complete VP1 sequences. The three sequences of TuPV (KM598418.1; KM598420.1; KM598421.1) were not used in the analyses. The possible recombination point is shown in the sequences from outbreak of JD (USP 507-1; USP 507-3; USP 507-9D; USP 507-20; USP 507-24); all sequences showed the same point recombination. Pink color = Track of sequence with a beginning and ending breakpoint of recombination. Yellow line = Mayor parent − minor parent; Turquoise line = Mayor parent − recombinant; Purple line = Minor parent − recombinant.

The first recombination breakpoint in the first part of the VP1 gene was generated between sequences of group III and group IV (JD) of ChPV. The first point of recombination had a beginning breakpoint in NT 1398 with a probability of 2203 × 10^−4^ and an ending breakpoint at NT 841 with a probability of 3.993436 × 10^6^, showing the minor parent of the sequence of ChPV USP 358-10 and the major parent unknown (USP 259-10). Both sequences belonged to group III of ChPV, where the reference sequence of ChPV ABU-P1 was included (Figure 2). The recombination points determined with SimPlot version 3.5.1 software are the same as those determined with RDP4 version v.4.43 software. The results of recombination in the sequences of JD showed that the possible origin of these sequences could be sequences of ChPV from group III from Brazil associated with RSS.

**Figure 3:**
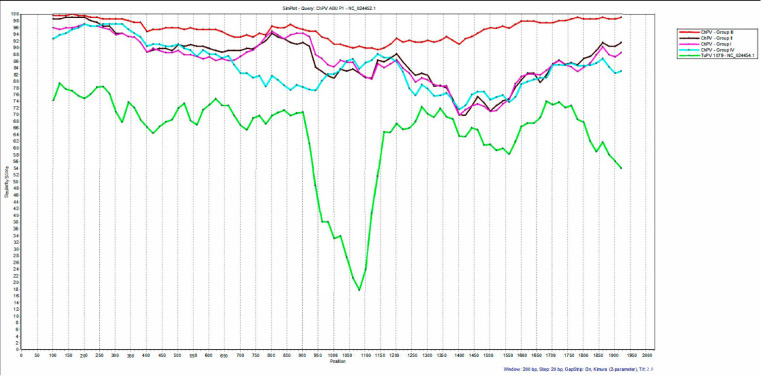
Simplot analysis of nucleotide sequences of ChPV based on full VP1 gene using ABU-P1 as the query sequence. The lines ChPV—Group I, ChPV—Group II, ChPV—Group III, and ChPV—Group IV represent each group of ChPV; Line ChPV—Group I includes sequences belong to Group 1 from Republic of Korea (KJ486489.1, KJ486490.1, KJ486491.1, KM254172.1); Line ChPV—Group II includes sequences belong to Group II from Brazil, China, United States, and Republic of Korea (KU523900.1, KM598416.1, KM254173.1, KU569162.1, KM598414.1, KM598415.1); Line ChPV—Group III includes sequences belong to Group III from Brazil of chickens affected with RSS here analyzed; Line ChPV—Group IV includes sequences belong to Group IV from Brazil of hens with JD studied in the present investigation; the green line showed the reference sequence of TuPV 1079.

The SimPlot analysis using sequences of each of the four clusters obtained in the phylogenetic tree, compared to the analysis using the ChPV reference sequence as a query, showed four lines corresponding to each group where group III of Brazilian RSS sequences have more similarity with the ChPV reference sequence ABU-P1 at some points than sequences of groups I, II, or IV. Groups I and II share less similarity than group III with the reference sequence, and group IV shows less similarity with the ABU-P1 sequence, which has a high similarity of NT (93–98%) with the reference sequence of ChPV between the first 400 NT, after which there is a diminished similarity of NT arriving until 71.5% (NT 1400) similarity of NT. The TuPV sequence had few similarities with the reference sequence and other sequences of ChPV, principally because position 900, with 73.1% similarity, decreases to 18% at position 1081. The VP1 gene also showed recombination signals in the same sites of the VP1 gene sequences determined previously by RDP4 from chickens with enteric problems, showing JD, which was generated between sequences from group III of ChPV that included the reference sequence (Figure 3).

## 4. Discussion

Chicken parvovirus is an enteric virus responsible for a wide range of enteric diseases related principally to outbreaks of diarrhea that results in dwarfish and weak animals and has a high economic impact in poultry [3,10,13,18,30]. Currently, ChPV has been identified in several countries around the world [2,7,20,21,24]; however, little genetic information on ChPV genes has been available in public databases. Therefore, the present article showed the molecular characterization of the VP1 gene from samples of ChPV belonging to outbreaks of RSS and JD, determining that Brazilian chicken flocks are also circulating genotypes of ChPV belonging to groups III and IV.

The ChPV genome is composed of a nonstructural protein (NS) and a structural protein VP that forms the viral capsid [1,6]. The genetic characterization of ChPV was carried out based on the sequencing of the NS gene [25]; however, this procedure was not capable of distinguishing sequences from ChPV and TuPV. Some studies could, however, distinguish between the sequences that have a codon terminal in the NS gene [26].

Several studies have determined the presence of genomes of ChPV with some differences in NT and AA compared with the reference sequence of ChPV (NC_024452.1) [7,8,24,26]. Thus, the comparative analysis of the percentage of NT and AA of the sequences studied with other sequences of the parvo VP1 gene available in the bank determined some differences between the sequences. By grouping the sequences as in the phylogenetic tree [7], it was possible to observe that group I includes sequences from Republic of Korea and group II includes sequences from China, Republic of Korea, Poland, and Brazil [KU569162.1; KM598415.1 (not included in this study), with a high similarity of NT and AA among them and less similarity of NT and AA compared with the ABU-P1 reference, respectively. In group III, where the reference sequence and the studied sequences from cases of RSS are included, there was a high similarity of NT and AA among the sequences. The last group, which included the sequences from JD, showed a similarity of 100% of NT and AA among the sequences and showed less similarity with group III, which included the reference sequence and included sequences from China (Table 3). The groups had few differences among them; therefore, genotyping based on the similarity of NT and AA could not be used to determine if a sequence belongs to a specific genetic group, meaning that phylogenetic analysis was necessary. The analysis of the similarity of NT and AA showed a 10 to 14% difference in relation to NT within group III, which included the reference sequence. The difference related to AA was approximately 7 to 9%, which is in accordance with other previously published results [7,8,24,31].

Group I included sequences of three genomes from chickens with cases of RSS from Korea that in previous studies were also classified in a separate group from the one that included the reference sequence (Group III) [8]. The sequences from Asia, specifically from Korea, showed a difference of NT (81.1–81.3%) and AA (93%) with ABU-P1, and were considered a novel ChPV based on the NT and AA analysis [8]. Similarly, in the present work, the sequences from JD, based on coding sequences and phylogenetic analysis, showed differences from all published ChPV sequences; however, the complete genome sequences are necessary to make comparisons of all genes and proteins. Group II is composed of sequences from Korea, the United States, China, and Brazil (not included in this study), all of which are related to outbreaks of enteric diseases. In Brazil, our group previously reported the presence of an isolate of ChPV belonging to group II (KM598415.1) associated with RSS [31], showing that ChPV groups II, III, and IV circulate in commercial chickens.

The primers used herein could amplify the complete VP1 gene, and, when cloned, the DNA plasmid allowed us to sequence the complete sequence of the studied gene and obtain trusted sequences. Their analysis allowed us to genotype the strains of ChPV and showed the presence of two well-differentiated groups, the majority related to the reference sequence ABU-P1 and the sequences from JD clustered in a different group (group IV) between analyzed sequences.

The analyzed samples in the present survey of chickens with enteric problems came from different states of Brazil where there are many farms for raising chickens. São Paulo state has most samples positive for ChPV, and this state includes the sequences belonging to group III and IV of ChPV described here. We showed that broiler chicken sequences had a greater similarity of NT and AA with the ABU-P1 reference sequence, and layer hens’ sequences showed a considerable difference (less similarity of NT and AA) when compared with ABU-P1 at some points of the sequence, as was reported in the SimPlot analyses (Figure 3). These differences in similarity of NT are in accordance with previous studies of ChPV genotyping [8]. Recombination of DNA plays an important role in rapid viral evolution and has important implications for disease evolution [32]. These events could cause transformations in the genome that could translate into new strains that escape the host immune response provided by a vaccine or could cause different effects in tissues or in the presentation of diseases [26,32,33]. Here, we showed sequences of ChPV with breakpoints of recombination, determined by several methods. These sequences were obtained from hens showing severe emaciation, pale mucosa, beak and claw depigmentation, and principally the presence of severe dilatation of the jejunum accompanied by the presence of diarrhea. This condition triggered the death of the animal. The presence of the abovementioned breakpoint of recombination could influence the aggregation of the mentioned sequences in a unique group without any other sequence of ChPV published in the GenBank platform, although there is no large difference in the concentrations of NT and AA among the sequences of ChPV.

The recombination analysis showed that the sequences of ChPV derived from cases of JD are recombinant sequences of group III ChPV. Similar events were reported in other studies. Strains of ChPV showed the presence of recombination points included in their genome from chickens showing enteric diseases in Poland [26]. Analyzing all the data, we could infer that the origin of the ChPV detected in animals suffering JD could be from a recombination of sequences of ChPV circulating in the Brazilian chicken flocks; nevertheless, the sequences showed high similarity with the sequences from China, which could indicate that Asia has strains with the same genetic features as the sequences from Brazil. The sequences from cases of RSS could have their origin in Europe, such as the reference sequence of ChPV ABU-P1, and this genetic diversity between strains of ChPV makes it necessary to continue studying the genome of ChPV in Brazil and in the rest of world. Unfortunately, in Brazil there is no information regarding how long the virus has been circulating among chicken flocks; although cases of enteric diseases were observed in Brazilian poultry thirty years ago, the lack of diagnosis of enteric viruses meant that they were not reported.

Currently, only a few strains of ChPV have been reported worldwide. The first report of this virus was made by Kisary in 1984 and was associated with enteric diseases in young chickens. The strain that was isolated was called ABU-P1 and was designated as the reference sequence. After that, several reports of the presence of virus have been published, showing associations with outbreaks of enteric diseases, principally RSS. However, the lack of complete genome sequences of ChPV has made it impossible to have adequate epidemiological understanding of this virus and good differentiation of genotypes of ChPV. For this reason, it is important that future studies begin to sequence the complete genome or complete genes to perform genetic analysis. VP1 could be used to genetically differentiate strains of the virus and to help determine host specificity among ChPV and TuPV [24].

## 5. Conclusions

In the present investigation, in addition to the ChPV of group II, as previously reported, we found two new genotypes of ChPV in Brazilian chickens. One group was associated with the reference sequence ABU-P1, where the cases of RSS are included, and the other genotype was found with sequences of ChPV from cases of JD. The JD sequences are somewhat different from other sequences published for ChPV. For this reason, more studies using NGS sequencing are needed to elucidate whether these sequences (JD) could be novel genotypes of ChPV and to determine whether this strain could be the viral agent responsible for jejunal dilatation.

## Figures and Tables

**Table 1:** Characteristics of samples.

Sample	Type of Bird	Age	Clinical Signs of Enteric Disease		Post Mortem Examination	Brazilian State	Molecular Diagnostic of ChPV
			Diarrhea	RSS	Jejunal Dilatation		
USP 93	Broiler	36 D	Yes	No	No	SP	+
USP 162	Broiler	40 D	Yes	Yes	No	SP	+
USP 238-1	Broiler	33 D	Yes	Yes	No	SC	+
USP 238-5	Broiler	18 D	Yes	Yes	No	SC	+
USP 259-10	Broiler	15 D	No	No	No	PR	+
USP 259-11	Broiler	15 D	No	No	No	PR	+
USP 336-7	Broiler	8 D	Yes	Yes	No	SC	+
USP 336-15	Broiler	8 D	Yes	Yes	No	SC	+
USP 336-16	Broiler	8 D	Yes	Yes	No	SC	+
USP 345-15	Broiler	17 D	Yes	Yes	No	SP	+
USP 358-7	Broiler	5 D	No	No	No	SC	+
USP 358-10	Broiler	8 D	Yes	Yes	No	SC	+
USP 362-7	Broiler	14 D	Yes	Yes	No	RS	+
USP 400-7	Broiler	14 D	Yes	Yes	No	SP	+
USP 401-3A	Broiler	14 D	Yes	Yes	No	SP	+
USP 507-1	Layer Hen	50 W	Yes	No	Yes	SP	+
USP 507-3	Layer Hen	50 W	Yes	Yes	Yes	SP	+
USP 507-9D	Layer Hen	50 W	Yes	Yes	Yes	SP	+
USP 507-20	Layer Hen	50 W	Yes	Yes	Yes	SP	+
USP 507-24	Layer Hen	50 W	No	No	Yes	SP	+
USP 710-1	Broiler	NI	Yes	Yes	No	SP	+
USP 711-1	Broiler	NI	Yes	Yes	No	SP	+

D = Day; W = Week; NI = Non Informed; SP = São Paulo; SC = Santa Catarina; PR = Paraná; RS = Rio Grande do Sul; + = Positive to presence of ChPV by PCR.

**Table 2:** Primers sequences, gene target of ChPV for VP1 sequencing.

Virus	Gene Target	Primer Name	Sequence 5′–3′	Amplicon bp	Assay	Reference
ChPV	VP1	VP2CD1-F	TGAAAATGAAAATCGAAGACAAAA	2289	PCR	This Study
		VP2CD1-R	GAGAAAGCAAGACTCTTTATTGAAA			

bp = base pair.

**Table 3:** Differences of Nucleotides and Amino Acids of sequences of ChPV from Brazilian chickens flocks with enteric problems and other sequences of same virus from other countries available in the GeneBank.

N.	Virus	Group	Sequences	% Amino Acid Similarity																																														
				ChPV																																										TuPV				GPV
				Group I				Group II									Group III																					Group IV												
				1	2	3	4	5	6	7	8	9	10	11	12	13	14	15	16	17	18	19	20	21	22	23	24	25	26	27	28	29	30	31	32	33	34	35	36	37	38	39	40	41	42	43	44	45	46	47
1	ChPV	Group I	KJ486489.1 Korea	-	100	100	100	98.2	98.5	98	96.5	96.5	96.5	96.7	96.8	96.5	93	92.8	92.8	91.1	93.3	93.1	93	93.6	93.4	93.4	92.8	93.3	93.4	93.1	92.7	92.4	95.5	92.8	92.1	93.6	94.3	92	92	92	92	91.8	92.5	91.8	91.7	79.7	79.7	77.9	79.7	28
2			KJ486490.1 Korea	99.6	-	100	100	98.2	98.5	98	96.5	96.5	96.5	96.7	96.8	96.5	93	92.8	92.8	91.1	93.3	93.1	93	93.6	93.4	93.4	92.8	93.3	93.4	93.1	92.7	92.4	95.5	92.8	92.1	93.6	94.3	92	92	92	92	91.8	92.5	91.8	91.7	79.7	79.7	77.9	79.7	28
3			KJ486491.1 Korea	99.8	99.8	-	100	98.2	98.5	98	96.5	96.5	96.5	96.7	96.8	96.5	93	92.8	92.8	91.1	93.3	93.1	93	93.6	93.4	93.4	92.8	93.3	93.4	93.1	92.7	92.4	95.5	92.8	92.1	93.6	94.3	92	92	92	92	91.8	92.5	91.8	91.7	79.7	79.7	77.9	79.7	28
4			KM254172.1 Korea	98.8	98.8	98.9	-	98.2	98.5	98	96.5	96.5	96.5	96.7	96.8	96.5	93	92.8	92.8	91.1	93.3	93.1	93	93.6	93.4	93.4	92.8	93.3	93.4	93.1	92.7	92.4	95.5	92.8	92.1	93.6	94.3	92	92	92	92	91.8	92.5	91.8	91.7	79.7	79.7	77.9	79.7	28
5		Group II	USP 362-3 Brazil	95	95	95.1	94.9	-	97.9	98.2	96.2	96.8	96.8	97	97.7	96.7	92.8	93	93	91.1	93.4	93.3	93.1	93.7	93.6	93.6	92.8	93.4	93.6	93.3	92.5	92.2	96	92.7	92.2	93.7	94.9	91.8	91.8	91.8	91.8	91.7	92.5	91.7	91.4	79.6	79.6	77.8	79.6	27,7
6			KM598414.1 USA	95	94.9	95	94.7	94.8	-	97.6	95.8	96.5	96.7	96.7	97	96.7	93.3	92.8	93.1	91.2	93.6	93.4	93.3	94	93.7	93.7	93.1	93.6	93.6	93.4	93.3	92.7	95.4	93.1	92.4	93.9	94.9	92.1	92.1	92.1	92.1	92	93	92.2	92.4	79.3	79.3	77.5	79.3	28
7			KU569162.1 Brazil	94.5	94.3	94.5	94.9	94.4	94.1	-	97.3	97	97.1	97.1	97.9	96.5	93.1	93.3	93.3	91.7	93.7	93.6	93.4	93.7	93.9	93.9	93.1	93.7	93.9	93.6	93.1	92.7	95.7	93	92.5	94	94.5	91.8	91.8	91.8	91.8	91.7	92.8	92	91.7	79	79	77.2	79	28
8			KM254173.1 Korea	92.3	92.3	92.3	92.9	91.7	91.2	93.3	-	95.4	95.2	95.5	96.2	94.8	92.7	92.8	92.8	92.2	93.3	93.1	92.7	93.1	93.1	93.1	92.7	93	93.1	92.8	92.2	91.8	94.8	92.5	93.1	93.3	93.6	93.1	93.1	93.1	93.1	93	92.7	93.7	91.7	78.8	78.7	77.1	79	28,2
9			KX133422.1 China	92.8	92.7	92.7	93.3	93	92.5	94.7	93.3	-	99.1	99.8	96.4	95.7	93.1	93	93.3	90.6	93.4	93.6	93.1	93.9	93.9	93.9	93.1	93.7	93.9	93.6	92.8	92.5	95.7	93	92.7	94.8	95.1	90.9	90.9	90.9	90.9	90.8	92	90.9	90.9	79.4	79.3	77.6	79.6	28
10			KX133424.1 China	92.7	92.7	92.7	93.2	92.9	92.5	94.7	93.2	99.5	-	99.2	96.4	95.5	92.8	92.7	93	90.5	93.1	93.3	92.8	93.6	93.6	93.6	92.8	93.4	93.6	93.3	92.7	92.2	95.4	92.7	92.4	94.5	94.8	90.8	90.8	90.8	90.8	90.6	92	90.9	90.9	79.1	79.1	77.4	79.1	28.2
11			KX133425.1 China	92.8	92.8	92.8	93.3	93	92.5	94.7	93.3	99.8	99.5	-	96.5	95.8	93.3	93.1	93.4	90.8	93.6	93.7	93.3	94	94	94	93.3	93.9	94	93.7	93	92.7	95.8	93.1	92.8	94.9	95.2	91.1	91.1	91.1	91.1	90.9	92.1	91.1	91.1	79.6	79.4	77.8	79.7	28.2
12			KM598416.1 USA	91.3	91	91.1	91.6	91.5	91.8	93.2	90.6	92.3	92.3	92.2	-	96.7	93	93	93.1	91.7	93.6	93.4	93.3	94	93.7	93.7	93.1	93.6	93.7	93.4	92.7	92.4	95.7	92.8	92.7	93.9	95.7	92.4	92.4	92.4	92.4	92.2	93	92	91.8	79.4	79.4	77.6	79.4	28.2
13			KU523900.1 China	92.1	91.8	92	91.9	91.7	92.6	92.2	89.3	90.7	90.8	90.8	92.3	-	94.8	94	94.3	92.1	94.2	94.6	94.8	94.3	94.9	94.9	94.5	95.1	94.9	94.9	94.5	94.2	94.9	94.6	93.1	93.3	95.5	93	93	93	93	92.8	93.9	92.5	93.1	79	78.7	77.2	78.8	27.9
14		Group III	NC_024452.1 ABU P1 Hungary	88.1	88.1	88.2	88.1	88	88.5	88.3	87.5	87.7	87.7	87.8	88.3	88.8	-	98.9	99.5	94.8	99.4	99.5	99.4	98.5	99.2	99.2	99.2	99.4	98.9	99.5	98.8	97.9	96.4	99.5	97.6	97.6	96.8	93.1	93.1	93.1	93.1	93	93.7	93	93.6	79	78.8	77.4	79.1	27.4
15			USP 93 Brazil	89.1	89.1	89.1	89.5	88.9	89.1	90.1	88.2	89	88.9	89.1	89.1	89.5	95.5	-	99.4	95.1	98.9	99.4	99.2	98	99.1	99.1	99.2	98.9	99.1	99.1	98	97.1	96.5	98.8	97.6	97.6	96.8	93.3	93.3	93.3	93.3	93.1	93.7	93.1	93.3	79	78.8	77.4	79.1	27.3
16			USP 162 Brazil	88.5	88.3	88.4	88.9	88.6	89.1	89.3	88	88.5	88.6	88.6	89.4	89.6	96.3	96.4	-	94.9	99.5	99.7	99.5	98.3	99.4	99.4	99.5	99.2	99.4	99.4	98.3	97.4	96.5	99.1	97.7	97.7	97	93.1	93.1	93.1	93.1	93	93.6	93.1	93.4	78.8	78.7	77.2	79	27.4
17			USP 238-1 Brazil	87.9	88	88	88	87	87.2	87.9	88.1	86.7	86.8	86.8	87.9	86.9	92.7	92	91	-	94.8	95.2	94.9	94.2	94.9	94.9	95.1	94.8	94.9	94.9	94.5	93.6	93.4	94.6	95.4	93.9	93.4	94.6	94.6	94.6	94.6	94.5	94.2	94.6	93.3	77.6	77.4	76	77.8	27.3
18			USP 238-5 Brazil	89.3	89.2	89.3	89.6	89.9	89.7	90.5	88.9	89.5	89.4	89.5	89.7	89.3	95.3	96.5	95.5	92.1	-	99.5	99.1	98.2	99.2	99.2	99.1	99.1	98.9	99.2	98.2	97.3	97	98.9	97.6	97.6	96.8	93	93	93	93	92.8	93.4	93	93.4	79	78.8	77.4	79.1	27.3
19			USP 259-10 Brazil	88.3	88.3	88.3	88.7	88.8	88.6	89.4	87.6	88.7	88.7	88.7	89.9	88.8	94.2	96	95.1	90.7	96.6	-	99.5	98.6	99.7	99.7	99.5	99.5	99.4	99.7	98.6	97.7	96.8	99.4	98	98	97.3	93.4	93.4	93.4	93.4	93.3	93.9	93.4	93.7	79.1	79	77.5	79.3	27.3
20			USP 259-11 Brazil	88.3	88.3	88.4	88.4	88.2	88.7	89	87.5	88.2	88.1	88.3	89.2	89.1	97.5	94.9	96.8	91.8	94.8	95.1	-	98.5	99.2	99.2	99.5	99.1	99.2	99.2	98.5	97.6	96.4	99.2	97.6	97.6	96.8	93.3	93.3	93.3	93.3	93.1	93.6	93.1	93.7	79.3	79.1	77.6	79.4	27.3
21			USP 336-7 Brazil	89.4	89.4	89.4	89.5	90.1	90.2	90.5	88.5	89.5	89.4	89.4	90.2	88.6	94.2	95	94.1	90.9	95.9	96.3	94.5	-	98.6	98.6	98.3	98.5	98	98.3	97.9	97	97.1	98.3	97	97.9	97.6	92.7	92.7	92.7	92.7	92.5	93	92.7	93	79.3	79.1	77.6	79.4	27.7
22			USP 336-15 Brazil	88.8	88.8	88.8	89.2	88.8	88.9	89.5	87.8	88.6	88.6	88.7	88.8	88.9	93.8	96.6	95.1	90.6	96.3	97.5	94.3	96.2	-	100	99.2	99.8	99.4	99.7	98.9	98	97.1	99.1	98	98	97.6	93.1	93.1	93.1	93.1	93	93.6	93.1	93.4	79.1	79	77.5	79.3	27.3
23			USP 336-16 Brazil	88.6	88.6	88.6	89	88.6	88.8	89.5	87.7	88.6	88.6	88.7	88.8	88.9	93.8	96.6	95.1	90.5	96.2	97.6	94.3	96.1	99.8	-	99.2	99.8	99.4	99.7	98.9	98	97.1	99.1	98	98	97.6	93.1	93.1	93.1	93.1	93	93.6	93.1	93.4	79.1	79	77.5	79.3	27.3
24			USP 345-15 Brazil	88.3	88.1	88.2	88.5	88.3	88.5	88.8	87.5	88.2	88.3	88.3	88.7	89.2	95.6	96.5	96.5	91.4	95.7	95.6	96.1	94.4	95.8	95.9	-	99.1	99.2	99.2	98.3	97.4	96.4	99.1	97.9	97.6	96.8	93.4	93.4	93.4	93.4	93.3	93.7	93.3	93.7	79	78.8	77.4	79.1	27.4
25			USP 358-7 Brazil	88.1	88	88	88.3	88	88.4	89	87.5	87.9	88	88	89	88.9	94.4	95.8	94.6	90.7	96	97.3	94.4	96	96.9	96.8	94.7	-	99.2	99.8	99.1	98.2	97	99.2	97.9	97.9	97.4	93	93	93	93	92.8	93.7	93	93.3	79	78.8	77.4	79.1	27.3
26			USP 358-10 Brazil	89.1	89.1	89.2	89.2	89.3	89.3	90.4	88.7	89.3	89.3	89.4	89.9	89.6	97.2	95.1	96.1	92.2	95.5	95.3	97.8	94.5	94.1	94.2	95.4	94.5	-	99.4	98.3	97.6	96.8	98.8	97.4	97.4	97	93.1	93.1	93.1	93.1	93	93.6	93.1	93.4	78.8	78.7	77.2	79	27.3
27			USP 362-7 Brazil	88.3	88.1	88.2	88.6	88.5	88.8	89.1	87.9	88.2	88.2	88.2	88.9	89.1	95.8	96.1	96.4	91.5	95.9	95.6	94.9	94.9	95.5	95.5	97	96.6	95.1	-	98.9	98	96.8	99.4	97.7	97.7	97.3	93.1	93.1	93.1	93.1	93	93.9	93.1	93.4	79	78.8	77.4	79.1	27.3
28			USP 400-7 Brazil	88.4	88.2	88.3	88.7	88.4	88.9	89.3	87.7	88.2	88.3	88.2	88.4	89.3	93.7	96	94.7	90.6	95.5	95.8	93.4	95	96.7	96.7	94.8	97.7	93.5	96.5	-	97.6	96.1	98.6	97	97	96.5	92.2	92.2	92.2	92.2	92.1	93.1	92.4	93	78.4	78.2	76.8	78.5	26.8
29			USP 401-3A Brazil	88.4	88.4	88.4	88.8	88.6	88.8	89.8	88	88.8	88.9	88.9	89.3	89	93.6	95.6	94.5	90.5	95.4	96.2	93.4	95	96.3	96.3	94.5	96	94.1	95.1	96.3	-	95.5	97.7	96.1	96.1	95.7	91.8	91.8	91.8	91.8	91.7	92.4	91.7	92.2	77.9	77.8	76.3	78.1	26.7
30			USP 710-1 Brazil	91.6	91.6	91.7	91.9	91.9	91.4	93.6	90.9	91.9	91.8	91.9	91.3	90.2	93.2	94.1	93	91.2	94.6	93.5	93.1	94.1	93.3	93.3	92.9	93.2	94.8	93.2	92.9	93.2	-	96.2	95.5	96.4	97.1	93	93	93	93	92.8	93.7	93	92.7	79.3	79.1	77.6	79.4	27.3
31			USP 711-11 Brazil	87.8	87.8	87.8	88.1	88.1	88.4	88.6	88.1	88.2	88.2	88.3	88.9	89.6	95.8	95.8	96.6	91.1	94.8	94.6	95.6	93.8	94.8	94.8	95.8	94.5	95.2	96	94.5	94.6	92.7	-	97.7	97.7	96.7	93	93	93	93	92.8	93.6	92.8	93.4	79	78.8	77.4	79.1	27.3
32			KX084401.1 China	87.6	87.4	87.5	88.1	87.3	87.6	88.9	88.1	87.7	87.8	87.8	88.5	88.4	92.6	94.3	93.8	90.5	93.9	94.4	92.4	93.6	94.8	94.8	93.9	93.6	92.5	94.3	93.8	94.1	92.2	93.5	-	97.9	96.4	93.9	93.9	93.9	93.9	93.7	92.8	93.1	92.2	79	78.7	77.4	79.1	27.4
33			KX133421.1 China	89	88.7	88.9	89.2	88.7	88.9	90.1	88.1	89.7	89.6	89.7	89.4	87.9	92.1	94.1	93.6	89.5	93.7	94	92.4	94.1	94.6	94.5	93	92.8	92.5	93.1	93	93.4	92.6	93.1	96.1	-	96.5	92.1	92.1	92.1	92.1	92	93.1	91.8	92.1	79	78.8	77.4	79.1	27.4
34			KM598415.1 USA	92.1	92	92.1	91.7	91.7	92.1	91.8	89	90.7	90.8	90.8	90.7	91.6	91.9	92.9	92.8	89.6	92.8	92.3	91.3	93.4	92.9	92.7	92.4	91.8	91.8	92.7	92.1	92	93.2	92.6	91.9	91.6	-	93.1	93.1	93.1	93.1	93	93.7	93	92.8	79.6	79.4	77.9	79.7	28
35		Group IV	USP 507-1 Brazil	86.9	86.9	86.9	87.1	86.4	86.9	86.6	89	85.9	86	85.9	87.2	85.7	86.5	87.1	86.3	91.4	87.6	86.4	86	86.7	86.5	86.4	86.7	86.3	86.7	86.2	86.1	86.2	87.4	86.1	86.3	85.5	86.8	-	100	100	100	99.8	98	97.6	95.8	78.8	78.5	77.1	79.1	28
36			USP 507-3 Brazil	86.9	86.9	86.9	87.1	86.4	86.9	86.6	89	85.9	86	85.9	87.2	85.7	86.5	87.1	86.3	91.4	87.6	86.4	86	86.7	86.5	86.4	86.7	86.3	86.7	86.2	86.1	86.2	87.4	86.1	86.3	85.5	86.8	100	-	100	100	99.8	98	97.6	95.8	78.8	78.5	77.1	79.1	28
37			USP 507-9D Brazil	86.9	86.9	86.9	87.1	86.4	86.9	86.6	89	85.9	86	85.9	87.2	85.7	86.5	87.1	86.3	91.4	87.6	86.4	86	86.7	86.5	86.4	86.7	86.3	86.7	86.2	86.1	86.2	87.4	86.1	86.3	85.5	86.8	100	100	-	100	99.8	98	97.6	95.8	78.8	78.5	77.1	79.1	28
38			USP 507-20 Brazil	86.9	86.9	86.9	87.1	86.4	86.9	86.6	89	85.9	86	85.9	87.2	85.7	86.5	87.1	86.3	91.4	87.6	86.4	86	86.7	86.5	86.4	86.7	86.3	86.7	86.2	86.1	86.2	87.4	86.1	86.3	85.5	86.8	100	100	100	-	99.8	98	97.6	95.8	78.8	78.5	77.1	79.1	28
39			USP 507-24 Brazil	86.9	86.9	86.9	87	86.3	86.9	86.6	89	85.9	86	85.9	87.1	85.7	86.5	87	86.2	91.4	87.6	86.3	86	86.6	86.4	86.3	86.6	86.3	86.6	86.2	86.1	86.1	87.4	86.1	86.3	85.4	86.7	99.9	99.9	99.9	99.9	-	97.9	97.4	95.7	78.8	78.5	77.1	79.1	28
40			KX133416.1 China	86.4	86.5	86.4	86.7	85.7	86.9	86.2	87.6	85.8	86	85.9	86.7	87.3	86.8	87.2	86.6	89.9	86.8	86.2	87	85.7	86.2	86	86.2	86.6	86.7	86.4	86.4	86.2	86.4	86.9	85	85.1	86.2	93.2	93.2	93.2	93.2	93.1	-	97.3	96.8	78.5	78.4	76.8	78.8	28
41			KX084400.1 China	86.2	86.3	86.2	86.3	85.5	86.3	86	89.2	85.7	85.8	85.8	85.9	85.7	86.1	86.5	85.7	89.9	86.5	85.7	85.9	85.7	85.9	85.8	86.3	85.6	86.1	86.1	85.3	85.6	86.2	85.9	85.3	84.6	86	93.4	93.4	93.4	93.4	93.3	92.9	-	97.4	78.5	78.4	76.8	78.8	28.3
42			KX133427.1 China	85.7	85.8	85.7	85.7	85	86.6	85.6	88.1	85.7	85.8	85.8	86.2	86.7	86.9	86.7	86.3	89	87.1	86.1	86.6	86.1	86	85.9	87	85.9	86.7	86.7	85.8	85.9	86	86.6	85	84.7	86.4	91.5	91.5	91.5	91.5	91.4	92.5	96.7	-	79.4	79.3	77.6	79.7	27.7
43	TuPV		NC_024454.1 Hungary 1079	73.5	73.4	73.5	73.5	73.4	73.3	73.5	73.1	73.6	73.5	73.6	73.5	73.3	73.3	73.4	73.3	72.4	73.9	73.5	73.3	73.7	73.5	73.5	73.2	73.5	73.2	73.5	73.6	73.5	73.5	72.9	73.2	73.1	73.7	73.3	73.3	73.3	73.3	73.4	72.9	73.3	74.3	-	99.4	98.2	99.2	26.7
44			30.KM598418.1 USA	73.5	73.4	73.4	73.5	73.4	73.1	73.5	73	73.6	73.5	73.6	73.5	73	73.3	73.2	73.2	72.4	73.9	73.5	73.4	73.7	73.5	73.5	73	73.6	73.2	73.4	73.5	73.5	73.6	72.8	73	73.1	73.5	73.3	73.3	73.3	73.3	73.4	72.8	73.1	74.2	98.8	-	97.6	99.1	26.8
45			31.KM598420.1 USA	73	72.8	72.9	73	72.8	72.7	73	72.5	73.1	73	73.1	73	72.7	72.7	72.8	72.8	71.8	73.3	72.8	72.7	73.1	72.8	72.9	72.6	72.9	72.6	73	73	72.9	73	72.4	72.6	72.6	73.1	72.7	72.7	72.7	72.7	72.8	72.3	72.7	73.7	99.3	98.1	-	97.4	26.5
46			32.KM598421.1 USA	73,3	73.2	73.2	73.4	73.2	72.9	73.3	73.1	73.6	73.5	73.6	73.4	73	73.2	73.1	73.3	72.4	73.7	73.4	73.4	73.7	73.4	73.4	72.9	73.5	73.2	73.3	73.4	73.4	73.5	72.8	72.9	72.9	73.4	73.2	73.2	73.2	73.2	73.3	72.8	73.1	74.3	98.7	98.9	98	-	26.7
47	GPV		NC_001701.1	46,4	46.3	46.3	46.3	46.1	46.9	46.7	46.5	46.7	46.8	46.8	47	46.7	46.3	46.8	46.2	46.3	46.5	46.9	46.4	46.8	46.9	46.9	46.5	47	46.4	46.6	46.6	46.3	46.7	46.5	46.3	46.6	46.7	46.4	46.4	46.4	46.4	46.4	46.2	45.9	45.8	44.6	44.3	44.6	44.3	-
				% Nucleotides Similarity																																														

## Data Availability

All data are included in this published article. The sequence data obtained in this study were uploaded to NCBI and the accession numbers can be found in the “GeneBank Accession Numbers” section.
